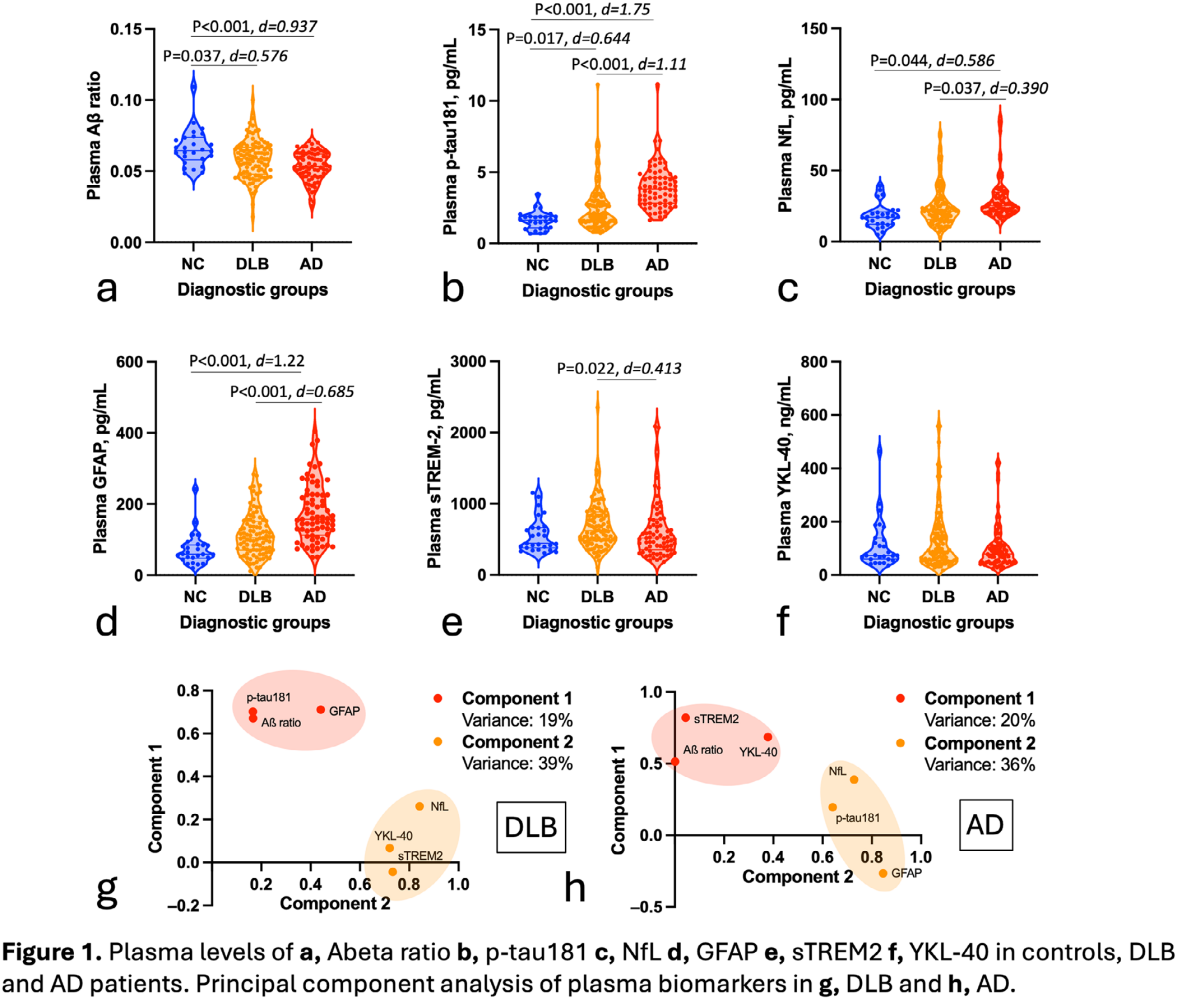# Plasma biomarkers in dementia with Lewy bodies: evidence from memory clinic settings

**DOI:** 10.1002/alz.084595

**Published:** 2025-01-09

**Authors:** Agathe Vrillon, Olivier Bousiges, Karl Götze, Catherine Demuynck, Candice Muller, Alix Ravier, Benoit Schorr, Nathalie Philippi, Emmanuel Cognat, Claire Hourregue, Julien Dumurgier, Matthieu Lilamand, Benjamin Cretin, Frédéric Blanc, Claire Paquet

**Affiliations:** ^1^ Assistance Publique‐ Hôpitaux de Paris, Centre de Neurologie Cognitive, Hôpital Lariboisière Fernand Widal, Paris France; ^2^ Inserm UMR S1144, Paris Diderot University, Paris France; ^3^ Hôpitaux Universitaire de Strasbourg, Laboratoire de Biochimie et Biologie Moléculaire, et CNRS, Laboratoire de Neurosciences Cognitives et Adaptatives (LNCA), Strasbourg France; ^4^ AP‐HP Nord, Université Paris Cité, Cognitive Neurology Center Hôpital Lariboisière‐Fernand Widal, Paris, Paris France; ^5^ Université Paris Cité, INSERM, UMRS 1144, Paris, Paris France; ^6^ University Hospital of Strasbourg, Geriatrics, Neurology & CMRR, Strasbourg France; ^7^ University Hospital of Strasbourg, CM2R (Memory Resource and Research Centre), Service of Gerontology Mobile‐Neuro‐Psy‐Research, Geriatrics Department, Strasbourg, Alsace France; ^8^ University Hospital of Strasbourg, CM2R (Memory Resource and Research Centre), Service of Gerontology Mobile‐Neuro‐Psy‐Research, Geriatrics Department,, Strasbourg, Alsace France; ^9^ University Hospital of Strasbourg, CM2R (Memory Resource and Research Centre), Service of Gerontology Mobile‐Neuro‐Psy‐Research, Geriatrics Department, Strasbourg, Strasbourg France; ^10^ Geriatrics and Neurology Units, Research and Resources Memory Center (CMRR), Hôpitaux Universitaires de Strasbourg, Strasbourg France; ^11^ University of Strasbourg & CNRS, ICube laboratory (UMR 7357), Strasbourg France; ^12^ INSERM UMR‐S942 Université Paris Diderot, Paris France; ^13^ Cognitive Neurology Center, GH Saint‐Louis ‐ Lariboisière ‐ Fernand‐Widal, APHP, Paris, Paris France; ^14^ Cognitive Neurology Center, Hôpital Lariboisière‐Fernand Widal APHP, Paris, Paris France; ^15^ University of Strasbourg and CNRS, ICube Laboratory UMR 7357 and FMTS (Fédération de Médecine Translationnelle de Strasbourg), IMIS team, Strasbourg, Strasbourg France; ^16^ Memory Resource and Research Centre, Neuropsychology Unit, Neurology Service, University Hospital of Strasbourg, Strasbourg, Strasbourg, Alsace France; ^17^ University of Strasbourg and French National Centre for Scientific Research (CNRS), ICube Laboratory and Federation de Medecine Translationnelle de Strasbourg (FMTS), Team Imagerie Multimodale Integrative en Sante (IMIS)/ICONE, Strasbourg France; ^18^ INSERM UMR‐S1144, Paris France; ^19^ Cognitive Neurology Center, Hôpital Lariboisière‐Fernand Widal APHP, Paris France

## Abstract

**Background:**

Increasing evidence supports the use of plasma biomarkers of amyloid, tau, neurodegeneration and neuroinflammation for diagnosis of dementia. However, their performance for positive and differential diagnosis of dementia with Lewy bodies (DLB) in clinical settings is still uncertain.

**Method:**

We conducted a retrospective biomarker study in two tertiary memory centers, Paris Lariboisière and CM2RR Strasbourg, France, enrolling patients with DLB (n=104), Alzheimer’s disease (AD, n=76) and neurological controls (NC, n=27). Measured biomarkers included plasma Aβ ratio, p‐tau181, NfL and GFAP using SIMOA and plasma YKL‐40 and sTREM2 using ELISA. DLB patients with available CSF analysis (n=90) were stratified according to their CSF Aβ profile.

**Results:**

DLB patients displayed altered plasma Aβ ratio, p‐tau181 and GFAP levels compared with NC and altered plasma Aβ ratio, p‐tau181, GFAP, NfL and sTREM2 levels compared with AD patients (Figure 1, a‐f). Plasma p‐tau181 best differentiated DLB from AD patients (ROC analysis, area under the curve [AUC]=0.80) and NC (AUC=0.78) and combining biomarkers did not improve diagnosis performance. Plasma p‐tau181 was the best standalone biomarker to differentiate amyloid‐positive from amyloid‐negative cases (AUC=0.75) and was associated with cognitive status. Combining plasma Aβ ratio, p‐tau181 and NfL increased performance to identify amyloid copathology (AUC=0.79). Principal component analysis identified different segregation patterns of plasma biomarkers in AD and DLB groups (Figure 1, g‐h).

**Conclusion:**

Amyloid, tau, neurodegeneration and neuroinflammation plasma biomarkers are modified in DLB, albeit with moderate diagnosis performance. Plasma p‐tau181 has potential contribute to identify Aβ copathology in DLB.